# Evaluating the holistic costs and benefits of corn production systems in Minnesota, US

**DOI:** 10.1038/s41598-020-60826-5

**Published:** 2020-03-03

**Authors:** Harpinder Sandhu, Nadia El-Hage Scialabba, Chris Warner, Fatemeh Behzadnejad, Kieran Keohane, Richard Houston, Daniel Fujiwara

**Affiliations:** 1 School of Natural and Built Environments, University of South Australia, Adelaide, Australia; 2 Food and Agriculture Organization of the United Nations, Rome, Italy; 3 Simetrica, London, UK

**Keywords:** Environmental impact, Environmental economics

## Abstract

Global agriculture aims to minimize its impacts on environment and human health while maintaining its productivity. This requires a comprehensive understanding of its benefits and costs to ecosystems and society. Here, we apply a new evaluation framework developed by the Economics of Ecosystems and Biodiversity for Agriculture and Food (TEEBAgriFood) to assess key benefits and costs on the production side of genetically modified (GM) and organic corn systems in Minnesota, USA. The market value of GM corn is $4.5 billion, and only $31.8 million for organic corn using production data and market prices of 2017. GM corn generates revenue of $1488 per hectare (at $121 per MT), which is significantly lower than the organic corn at $2793 per hectare (at $294 per MT). Using a novel three-stage wellbeing valuation, analysis of the associations between corn production intensity and subjective measures of general health and wellbeing indicates that the total non-financial health cost associated with GM corn is $427.50 per hectare or $1.3 billion annually. We also find that the total annual environmental cost associated with GM corn production is $179 per hectare or $557.65 million within Minnesota. The use of the evaluation framework can help to improve decision making at farm and policy level to develop sustainable agriculture in order to minimize environmental and health related costs to society and economy.

## Introduction

Meeting the food demand of increasing human population requires increased production and also a major policy shift in the way food is produced, processed, distributed and consumed^1–3^. Another key challenge of global agriculture is to minimize impacts on environment and human health^4,5^. Agriculture occupies 38% of land worldwide, its contribution to Gross Domestic Product (GDP) is less than 1% in developed countries and up to 50% in some developing countries^6^, and it produces sufficient calories to meet the current food demand of human population^7^. However, 815 million are undernourished worldwide^7^. At the same time, 2.1 billion people are overweight and adult obesity is on the rise, which is a major risk factor for non-communicable diseases, such as cardiovascular disease, diabetes and some cancers^7^. These non-communicable diseases have high economic costs to individuals, societies and the governments. One-third of the agricultural produce is wasted during harvesting, processing and consumption^5^. Agriculture accounts for one-fifth of the global greenhouse gas emissions. Annually, 145 million tonnes of synthetic fertilizers are applied in agriculture along with pesticides and veterinary chemicals. These agrochemicals, along with some high impact agricultural practices and high fossil fuel energy use, have resulted in pollution of water ways, eutrophication, depletion of freshwater resources, increased greenhouse gas emissions, land degradation and loss of biodiversity^8,9^. In economic terms, these impacts are often known as negative externalities. Agriculture also produces many benefits to human society in the form of food, feed, fibre, bio-products, maintenance of genetic material, carbon sequestration, landscape aesthetics, recreational opportunities, etc., which are widely known as ecosystem services and increasingly being studied in agricultural systems^10,11^. These are considered as positive externalities in agriculture. However, the current global economic system does not capture any negative impacts such as damages to environment and human health, or benefits in the form of ecosystem services, which are linked to agriculture and food sector^5^. Therefore, the society and economy are unable to perceive any hidden costs or benefits of agriculture and food systems. This often leads to pervasive outcomes such as high costs to society and the environment. One way to address this issue is to assess all positive and negative externalities associated with agricultural production systems in order to help develop appropriate response to shift farm practices and policies towards sustainable agriculture and protect environmental and human health. As a case study to demonstrate proof of concept, we focused on corn in Minnesota, USA, and analysed key externalities associated with the production side of genetically modified (GM) and organic corn systems using true cost accounting (TCA) method by following TEEBAgriFood evaluation framework (The Economics of Ecosystems and Biodiversity for Agriculture and Food)^5^.

TEEBAgriFood is a United Nations Environment led initiative that has developed a common universal and inclusive framework known as the TEEBAgriFood evaluation framework to evaluate all significant externalities of food production systems across the value chain. It aims to understand and value links between natural, social and human capital in agriculture and food systems more holistically and reflect them in an economic system by evaluating true costs and benefits^5^. It intends to develop appropriate policy response to support the growing demand for diverse and nutritious food with less damages to environment and human health.

Corn plays an important role in the global economy, with USA being the leading producer of 370 million tonnes from 36 million hectares (harvested 33.08 million hectares in 2017), which accounts for over one-third of the global corn production^12^. Out of this, more than 92% is GM corn. Currently, with global production of 1.06 billion tonnes from 187 million hectares, it is second to sugarcane and in global trade, it is the second most traded agricultural commodity after wheat^12^. In industrialised countries, it is mostly used as animal feedstock followed by ethanol and other industrial uses. Whereas, in other countries, most of the corn is used directly for human consumption.

Application of unnecessary large inputs of ammonium and nitrate fertilizers and herbicides in corn production has become a major source of various kinds of pollution such as water pollution by fertilizer run off into rivers and streams, which leads to hypoxic, oxygen-deprived areas, where aquatic life cannot survive^13^. This has been a major challenge in the Mississippi river basin as it flows into the Gulf of Mexico^14^. It is established that 40% of the nitrogen pollution that contributes to this comes from fertilizer application in corn as it requires relatively high levels of nitrogen^15^. Similarly, a rising nitrate level in drinking water is also linked to high fertilizer and pesticide application in the corn growing regions in US^16^. For example, harmful algal blooms in Lake Erie; unsafe levels of nitrate in the rivers Des Moines, Iowa; high nitrate levels in two municipal wells in Randall, Minnesota. Industrial scale corn production also requires large amounts of fossil fuel inputs for cultivation, harvesting, drying and transport, which contributes to greenhouse gas emissions^17^. Excessive use of nitrogen fertilizers in corn also contributes to the rising atmospheric levels of nitrous oxide (N_2_O)^18^. Corn monoculture has also promoted losses in terms of crops and genetic biodiversity of arthropods and other fauna^19^. The impacts of corn are not only limited to the natural environment – it also significantly affects human health^20,21^. However, GM corn can also have positive impacts on non-GM corn production and the economy. For example, use of GM corn resulted in an area wide pest suppression with combined economic benefits of $3.2 billion to corn growers in Illinois, Minnesota, and Wisconsin states, over a period of 14 years^22^.

Therefore, this study assesses two systems – a dominant GM corn production system and organic system that covers a small fraction of the total area under corn in Minnesota, USA. We estimate various costs and benefits of GM and organic corn systems in the State of Minnesota, USA by applying the TEEBAgriFood evaluation framework. The main contribution of this study is an example of the application of the TEEBAgriFood framework in agricultural production systems. The study advances the use of true cost accounting methods to support policy on sustainable agriculture, in order to minimize environmental and health related costs to society and wider economy.

## Results

Here we report results of the assessment of two diverse types of corn production systems in terms of stocks and flows of four capitals.

### Produced capital

Here we summarize key outputs in two production systems – GM corn and organic corn (Table 1). Organic acreage is less than 1% of the total corn area in the year 2017. The yield is significantly lower in organic production system. However, the market price, in 2017, of organic corn is 2.4 times more than GM corn.

**Table 1:** Acreage, total production, market price of GM and organic corn in Minnesota, in the year 2017.

	GM corn	Organic corn
Area harvested, million ha	3.04	0.01
Average yield, MT per ha	12.3	9.5
Total production, million MT	37.4	0.1
Market value of corn, $ million	4510	31.8
Average price of corn per MT	121	294

The costs, in 2017 USD, of fixed and variable assets on a typical corn farm, are summarized in Table 2. Corn yield based on data obtained from USDA show higher yield in GM corn than the organic corn. However, net returns are found to be higher in organic corn in two corn growing regions (Table 2).

**Table 2:** Organic and GM corn production costs and returns (in 2017 USD) per planted hectare, by region in 2010 (Data from USDA ERS 2014).

	Organic $/ha		GM $/ha	
	Heartland	Northern Crescent	Heartland	Northern Crescent
Total, gross value of production (corn grains and silage)	2268	2149	1917	1841
Total, Operating costs (seed, fertilizer, chemicals, fuel, electricity, interest on operating capital, repairs)	455	619	775	773
Total overhead costs (hired labour, opportunity cost of unpaid labour, capital recovery of machinery, opportunity cost of land, taxes and insurance, general farm overhead)	856	761	741	590
Total costs	1310	1380	1516	1362
Value of production less total costs	957	770	400	479
Value of production less operating costs	1813	1531	1142	1068
Yield (Metric Tonne per ha)	8	7	11	10
Price ($ per Metric Tonne of corn grains at harvest)	298	299	181	184

### Social capital

In Minnesota, corn growers have an extensive network that extends from individuals to community and from farm level to national level (Tables 3 and S4, Supplementary Information). This network extends in both private and public sectors of the corn-based economy in US. There are three main dimensions of social capital – structural, relational and cognitive. The structural dimension is the pattern of connections and networks among actors and includes bonding, bridging and linking of social interactions^23^. Bonding is interaction between members of a relatively homogenous group (family or close friends), while bridging refers to the interconnections between heterogeneous groups (agri-businesses, farming groups etc.). Ties between individuals, or the groups they belong to, etc., are known as linking social capital.

**Table 3:** Social networks available to corn growers in Minnesota.

Networks	GM corn growers	Organic growers	Informal/Formal/Transactional
Government	26	26	Informal/Formal
Research	8	11	Informal
Farming/environment groups	18	17	Informal
Businesses	8	6	Transactional
Individuals (Friends/neighbours/community)	2	2	Personal
Foundations and non-profits	7	7	Informal
Total	69	69	

### Human capital

To understand the type and form of human capital associated with corn production systems in Minnesota, we provide a snapshot of the various aspects of the rural population. In 2017, 1.22 million Minnesotans live in rural areas, which represents 22% of Minnesota’s total population of 5.57 million. Since 1900 there has been a continuous downward trend in the rural population due to migration to urban areas. Out of 1.22 million rural population, there are about 73,400 farmers in Minnesota. The average age of famer is more than 55. About 8.5% are women operators. In terms of highest qualification levels achieved, the majority of rural Minnesotans have a high school qualification, whilst the majority of urban and town dwellers have a Bachelor’s degree or above.

### Valuation of the non-financial health costs associated with corn production

The non-financial health costs are the costs to an individual’s wellbeing, which are predominantly realised through subjective health impacts associated with corn systems and are not typically accounted in the farm or national accounts. The valuation of non-financial health costs of corn production is based on three-stage well-being valuation method (Methodology for Valuing the Agriculture and the wider food-system Related Cost of Health, MARCH)^4^. This method monetises the impact of corn production by equating the change in life satisfaction, as a measure of well-being, from a marginal increase in corn production to the change in income required to yield an equivalent change in well-being. We first linked satellite data on agricultural land use to measures of general health from the Gallup Daily tracking survey 2018–2017. We then used the well-being valuation method to measure the association between corn production near individuals’ residences and their general health in Minnesota. The costs in 2017, associated with a 1% increase in corn intensity in the vicinity of an individual’s residence is $20.70 per year when considering a 5 km buffer around each respondent’s home address ZIP code and $24.70 per year in the 10 km buffer. These results are based on the average annual household income in Minnesota in 2016, which, according to the US Census Bureau, was $83,100.

The total non-financial health costs in Minnesota are obtained by aggregating all counties health costs. Based on our estimates, the annual non-financial health costs of corn production in Minnesota are $1.3 billion ($233 per capita per year or $427.50 per hectare per year).

While our results show a statistically significant association between corn intensity in the proximity of individuals and their health, we cannot determine the channels through which this relationship is realised. Water and air pollution from corn production is one possible explanation but quantifying the specific channels through which corn intensity affects health requires further exploratory analysis.

The health costs estimated here are based on the production side of the corn value chain, linked to the corn intensity effect on environmental quality. These non-financial health costs do not include capital costs incurred in the public health system, loss of economic productivity, and loss of taxes and GDP. They may, to a certain extent, include the individual medical expenditures associated with the health impacts of living near corn farms, although this cannot be confirmed with the data available.

Regarding organic corn production, there is some evidence of the reduced adverse health impact of corn intensity associated with the presence of local organic production. However, a more rigorous analysis of the impact of organic production would require access to the exact planted area (or total yields) of all organic farms within each ZIP code. This may become available if the prevalence of organic corn farming increases over time.

### Natural capital

We provide an estimate of the benefits (ecosystem services) and costs, in 2017 USD, associated with GM corn cultivation in terms of impacts on climate change, water quality, air quality, and soil quality (Table 4).

**Table 4:** Environmental costs in GM corn production system in Minnesota.

Natural Capital Change	Indicator	Unit quantity and type	Marginal social cost (2017 $)	Net (+/−) benefit (2017 $)
Climate Change	CO_2_ emissions from N fertilizer production	1,570,995 Mg CO2e (392,748,819 kg N x 0.004 Mg CO_2_e per kg N fertilizer production)	42.55 per Mg CO_2_e Emissions in 2015 assuming a 3% discount rate	Statewide: −66,850,863 Per hectare of corn: −21.47
Climate Change	N_2_O emissions from N fertilizer application	392,748,819 kg N fertilizer application to corn	0.235 per kg N Assuming a 3% discount rate	Statewide: −92,316,643 Per hectare of corn: −29.65
Water Quality	Increased groundwater nitrate concentrations from leaching of N fertilizer	392,748,819 kg N fertilizer application to corn	0.075 Median cost of exposure of NO_3_- per kg N	Statewide: −29,285,663 Per hectare of corn: −9.41
Water Quality	Increased phosphorus loading in surface waters	1,991,320 kg P per year from corn production	55.43 per kg P	Statewide: −44,774,633 Per hectare of corn: −14.38
Air Quality	Premature mortalities caused by particulate matter 2.5 emissions from N fertilizer application	392,748,819 kg N fertilizer application to corn	0.55 Median cost of exposure of PM2.5 per kg N	Statewide: −216,633,669 Per hectare of corn: −69.59
Soil Loss	Damage to infrastructure, recreation, and business from sediment runoff. Soil productivity from loss of topsoil.	14.2 Mg soil loss per ha of corn production per year	5.93 per Mg Estimates for corn belt region	Statewide: −107,784,217 Per hectare of corn: −34.62

Organic corn and conventional corn are rarely studied with comparable practices. Cover crops and diverse, multi-year, rotations were commonly used in organic systems, in contrast to a two-year corn-soy rotation in a conventional system. Due to these differences, we found few instances where we could make definitive quantitative statements about the differences between these systems with regards to the indicators in this analysis.

Total environmental cost associated with GM corn production is $179 per hectare or $557.65 million annually in Minnesota. However, the range of estimates in the underlying studies was very large. Environmental costs estimated here are based on the production side of the corn value chain, linked to the inputs in corn production and do not include environmental costs associated with the transport, processing, and consumption.

**Figure 1:**
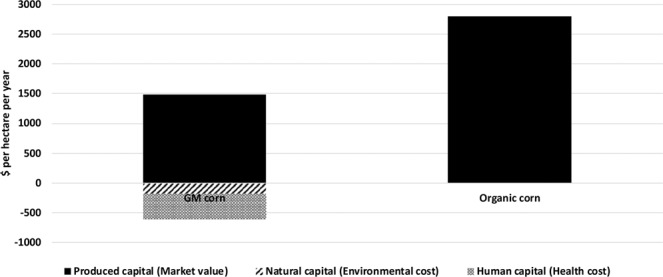
True cost of GM and organic corn production in Minnesota, US in 2017.

Given the data and information presented in this section, we provide a comparison of the true cost of corn production in Fig. 1. It includes produced capital, environmental and health cost of GM corn per hectare in Minnesota in 2017. For organic corn, the data shown is produced capital only.

## Discussion

We discuss above results in terms of the impacts both positive and negative by corn production on four capitals, policy drivers, implications for TEEBAgriFood evaluation framework and it’s limitations.

Corn is one of the major crops that contributes significantly to the gross domestic product (GDP) in Minnesota. Minnesota state GDP is about $368 billion, where agriculture contributes about 1.9% ($7 billion annually). Economic contribution from corn also includes other allied goods and services that supply all farm inputs, research, market support, finance and insurance, animal feed and ethanol production. Thus, corn is an important crop for the economy of Minnesota. The GM corn price varies between $150–180 per MT with peak price in 2012 at $333 per MT. These prices are subject to global demand and supply for corn-based animal feed. However, it is important to consider the total contribution of GM corn to the state economy as well as the USA economy. Organic corn prices are always higher than the GM corn at about $190–300 per MT due to its growing demand and low production. In 2012, organic corn price increased to $670 per MT.

The analysis presented here includes all GM corn varieties and does not differentiate GM corn on the basis of differences, such as Ht (herbicide-tolerant), Bt (*Bacillus thuringiensis*, insect-resistant), and Ht-Bt (stacked) varieties. Out of the total GM corn grown in USA, 80% are stacked gene varieties, 10% Ht varieties and about 10% conventional non-GM, hybrid varieties. There is need to treat transgenic varieties on a case-by-case basis to assess their impact on the environment and health. We have calculated the extent of corn production in an individual’s vicinity using satellite data from USDA which does not allow differentiation based on variety, GM vs. non-GM etc., which would require data linking to locational data on individual farms. For organic farms, we have attempted to do this using data from the USDA’s Agricultural Marketing Service. As organic farms represent a very small proportion of overall production, we do not have a large enough sample of individuals in the Gallup survey data to be able to separate impacts based on proximity to organic vs. non-organic farms. To do so would require a large sample survey data, or US-wide analysis of existing data.

Given higher net returns from organic corn, a greater number of GM corn farmers should convert to organic. However, organic practices are not being widely adopted as is evident from the 0.3% area under organic corn in Minnesota in 2017. There are a number of barriers such as the technology required for weed control, organic seed availability, market, insurance etc., which prevent mass scale conversion to organic farming.

The observed differences between GM and organic corn in production (Table 2) could be due to scale of farms. GM corn is grown on large scale farms, whereas, organic corn is mostly part of mixed farming systems and in rotation with other cereals and pastures (Table S1, Supplementary Information). More than 92% of corn in Minnesota is GM corn, therefore, the data obtained from the databases did not allow to differentiate between GM and conventional corn. However, given the scale of farming, in both GM and conventional corn production systems, it is likely that there are more similarities in terms of productivity and prices. In contrast, organic farms are fewer and are small scale as compared to GM corn farms (Table 1). The differences observed in production could be attributed to the nature of farming system.

There are both formal and informal social networks available to corn growers in Minnesota as summarized in Table 3 that add to the social capital related to agriculture in general and corn systems in particular. Some of these networks provide benefits to individuals such as neighbours, friends etc., while others provide group benefits^23^. Informal networks between neighbours, friends, grower groups are used to acquire training from others who have already adopted new practices. One example is the cover cropping group, which is a group of farmers that have adopted cover cropping in corn-soybean rotation to improve soil heath. Whereas, formal networks can help obtain assistance to implement various practices through extension activities, participation in conservation programs etc. These networks also help facilitate employment and market opportunities^24^. However, we did not study the efficacy of these networks in Minnesota. It will be useful to identify those networks that are more promising and effective in bringing positive change in corn production systems and make it financially and environmentally more sustainable.

For the human health cost related to corn systems in Minnesota, we considered official data (Gallup Daily Survey 2008–2017; https://www.gallup.com/home.aspx) that includes general health and disability data. Cancer is the leading cause of death in Minnesota, followed by cardio-vascular diseases, unintentional injury and chronic lower respiratory diseases. The result demonstrates that general health of individuals decreases by 0.67% with corn production in the respective zip code, totalling annual non- financial health costs of corn in Minnesota to $ 1.3 billion. The attribution of causation to individual diseases is highly challenging and whatever scientific studies are considered, results remain debatable. The methodological approach adopted in this study considered health outcomes associated to corn production (i.e., environmental quality) within Minnesota (e.g., not the entire Mississippi drainage basin), thus excluding eventual corn consumption impacts.

For the natural capital impacts of corn, environmental costs associated with production of corn are estimated in this study and do not include environmental costs associated with transport, processing, and consumption. While included variables incorporate most of the key factors that influence the environmental cost of corn production, the inclusion of additional factors or refinement of those evaluations could increase or decrease estimates of the net social cost of conventional corn production. The uncertainty remains high for such estimations. For example, the plausible social costs to drinking water, air quality, and N_2_O derived climate change, from 1 kg of N fertilizer applications ranged from $0.05 to over $10^25^. Using the assumptions presented above, the state-wide social cost could range from $19.6 million to $3.9 billion for just those metrics.

The environmental cost varies spatially. For example, production upwind of population centers has greater air quality costs caused by more people being exposed to PM2.5 emissions. Groundwater nitrate contamination risk is heavily influenced by the geology of the region, and the change in water clarity in response to the same amount of P loading varies from lake to lake. For these reasons, applying the costs presented here to other regions will not reflect the local social costs of corn production.

Our analysis does not consider the impacts of production and land use change in a global economic market context, which would require a host of assumptions about market responses and other factors.

Organic practices have a slight negative impact on corn yield. Organic corn has less CO_2_ emissions than conventional corn but similar N_2_O and CH_4_ emissions^26^. The reduced CO_2_ emissions come from the lack of synthetic N-fertilizer production. However, if more land is required to meet demand under organic production, land use change could negate these benefits^27^. The primary difference between the two systems with regards to nitrate leaching is conventional systems typically use synthetic fertilizer, which is more water soluble and can create runoff more easily than manure used in organic systems that is mixed in with the soil^28^. However, studies have found both that organic systems leach less^29^ and there is no differences in leaching between conventional and organic systems. More research is required to understand the magnitude of differences in leaching between the systems. The use of manure as a fertilizer source may provide more P than is needed to achieve maximum yields on Minnesota soil. However, no studies quantifying the P export of organic systems were reviewed. The average price of organic corn is higher ($284 per MT for organic versus $182 per MT for conventional in 2010). However, these prices reflect a much lower supply of organic corn relative to conventional (approximately 0.3% of corn production in MN is organic). One study found lower NOx emissions in a no till system compared to tilled system^30^. Increased tillage required for weed control in organic systems may result in greater NOx and subsequently greater PM2.5 emissions, however, research specifically comparing the precursors to PM2.5 emissions between conventional and organic systems was not found. While soil loss has been studied in conventional tillage and no-till systems, comparisons for conventional and organic were not found. As with air quality, the reliance on tillage for weed control in organic systems could result in more soil loss, but these differences have yet to be quantified.

Organic standards include a limited number of synthetic substances, which are not innocuous but approved by legislators for limited use. The USDA National List of synthetic substances includes materials that are allowed in organic crop production under certain circumstances. The list includes algaecides, disinfectants, sanitizers, irrigation system cleaners, herbicides, animal repellents, insecticides, miticides, pheromones, rodenticides, slug baits, plant disease controls, soil amendments, and plant growth regulators. USDA Organic Standards allow a total of 25 synthetic plant protection products (as compared to 900 for conventional agriculture) to keep farms economically viable in the absence of natural alternatives. Among which copper sulphate is used by many organic farmers. Copper sulphate is used as fungicide in organic orchards and not on organic grains, such as our corn fields. However, there is need to analyse impacts of each chemical in organic systems on health and the environment.

Market forces linked with US federal policy have driven corn production in Minnesota and throughout the Midwest. While corn has been major commodity in the region for decades, recent policy changes to the Farm Bill and the enactment of the Renewable Fuel Standard have protected and incentivized corn production by subsidizing insurance for corn production and mandating production volumes of corn-based ethanol. The US Farm Bill originated in 1930s and is regularly updated to address a wide range issues related to food and agriculture. In 2014, crop insurance subsides were expanded for corn and other crops, reducing the risk producers face from planting commodity crops on marginal land^31^. Demand for corn was bolstered with the Renewable Fuel Standard, a federal law designed to increase demand for agricultural commodities by mandating production of both corn and cellulosic based ethanol^32^. While corn production has been sufficient to keep pace with the corn ethanol volumes called for in the law, cellulosic production has not met targets. In addition to increased demand for corn, reductions in funding for the US Conservation Reserve Program have resulted in conversion of hundreds of thousands of acres of retired land to corn production^33^. These policies contributed to record corn production expansion in the US, both through crop switching and expansion on to marginal land^34^.

The TEEBAgriFood evaluation framework applied here to corn production is an opportunity to describe and monetize key positive and negative impacts in Minnesota. Although the framework prescribes to capture significant impacts throughout the value chain, it is a challenging task for the researchers to gather and assemble a large amount of data into the framework template to include all aspects of farming systems, society and the environment. Therefore, we focused on the production side of the corn systems only. The analysis does not only focus on quantitative data, it included descriptive information, monetary and non-monetary information, spanning from natural sciences to social and health sciences including economic values. Therefore, a multi-disciplinary team is required to undertake such an assessment.

The assessment is based on existing data and information, which may be a limiting factor in understanding the comprehensive costs and benefits. Access to wide set of resources is required for complete and meaningful assessment that can be used for making policy responses. There are several indicators related to corn production that did not have reliable data or information to be used in this assessment. Current databases are of limited use in terms of the analysis, as they don’t have individualized information about production, area, market prices of different types of varieties, for example, Bt, Ht, stacked, different organic varieties etc.

The framework itself is limited use in terms of comparing two systems, where there is data limitation. This study compared two dominant corn production systems and did not examine other stages of the corn value chain. The framework is useful in assessing macro level data that is required for policy analysis. However, systems level analysis requires much granular data that should include – types of varieties, farming systems, cropping rotations, time period, impacts of all chemicals and practices in different systems etc.

The assessment should not be interpreted as final estimation of all costs and benefits but as a pointer towards significant externalities (including magnitude of their costs to society and economy) that are unwarranted and are the result of current practices and policies. The assessment can be used as a source to review wider impacts of the entire corn value chain in order to modify policies and practices.

The health costs of corn production are significant as compared to the farmgate value of corn (Fig. 1). However, these are under-estimations of the true cost of corn to human health due to the exclusion of corn consumption and linked health costs, which are not being investigated in the study. Research on health impacts of corn systems provides tentative evidence for a potentially positive effect of organic corn systems, as compared to conventional corn operations. However, more research is required, with finer resolution data than district level data, including detailed locations of survey respondents and planted areas of organic production in order to estimate the health costs of organic corn. Granular data would also facilitate the development of an improved causal framework, affording future research increased confidence in its findings, and offering deeper insights. Expanding the analysis to include other corn-producing states would provide evidence as to whether the negative health effects of corn production hold on a broader scale, and in doing so increase sample size available to researchers.

## Conclusions

The study used the TEEBAgriFood framework and revealed high hidden social, environmental and health related costs associated with GM corn production systems as compared to organic production in Minnesota. The use of the evaluation framework can help to improve decision making at farm and policy level.

The results from the analysis suggests a positive influence of both systems on produced and social capital in Minnesota. For GM corn production systems, there are positive economic impacts, however, the divide between small- and large-scale farmers is increasing, leading to negative social, health and environmental impacts. GM corn is used for producing ethanol as it is supported by the current energy policy. For organic production systems, there are positive economic, social, health impacts, while limited environmental impacts. Practitioners can use this information to make a decision about production system that can improve farm practices. Whereas, policy makers can use this information to support systems that improve social, economic and environmental sustainability. However, this require a major shift in US agricultural and energy policies that support the current GM corn systems.

This multi-dimensional assessment has helped to understand significant impacts and dependencies and holistic costs and benefits of two production systems, however, it is vital to understand how farmers adopt this new information. There is need to develop pathways for change in consultation with the farming community with appropriate support from policy to effectively contribute towards the improvement in the environment and the well-being of society.

## Methods

### Study area

**Figure 2:**
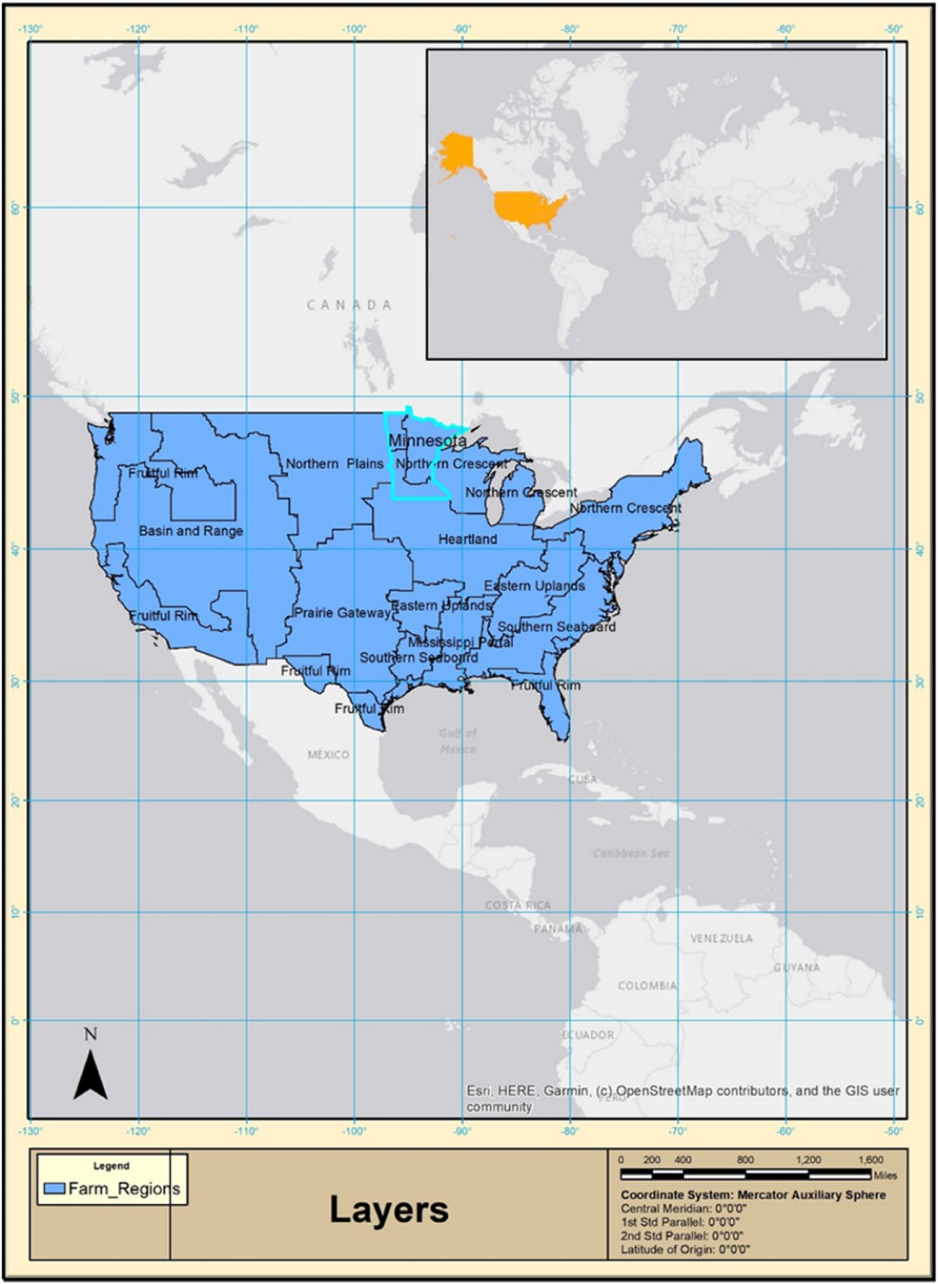
U.S. farm resource regions and the study area (Source: USDA ERS, 2010; Map generated by using Esri’s ArcMap software, version 10.6.1, https://desktop.arcgis.com/en/arcmap/10.6/get-started/main/get-started-with-arcmap.htm).

In the USA, the total area used to plant corn in 2017 was 36.04 million hectares (harvested 33.08 million hectares), with an average yield of 11.2 metric tonnes (MT) per hectare. The total value of corn was $48.46 billion at an average price of $130 per MT^35^. Minnesota falls under two regions - heartland and northern crescent (Fig. 2). Minnesota is the fourth largest corn producer in the USA, with 3.22 million hectares used to plant corn, of which3.04 million hectares is harvested, yielding an average of 12.3 MT/ha (range between 8.3–13.8 MT/ha). More than 92% of this corn is genetically modified (GM) and rest is hybrid^36^. There are three dominant types of GM/transgenic corn varieties used in US – Herbicide-tolerant (Ht) corn, *Bacillus thuringiensis* (Bt) corn and Stacked (Ht & Bt) corn. Ht varieties herbicide tolerant and offer weed control option to corn growers. Bt corn is insect-resistant and provide protection against the corn rootworm, the corn earworm and the European corn borer. Stacked varieties comprise both Ht and Bt traits and are being increasingly used since 2007^36^.

GM corn farming systems are described as a monoculture in rotation with GM soybean with high inputs of synthetic fertilizers and herbicides. GM and hybrid corn are grown primarily for ethanol production, where, Dried Distillers Grains (DDGs) are a by-product used as animal feed for livestock or poultry, and the meat products are consumed by humans. GM/hybrid corn is either grown in rotation with soybean or two subsequent corn crops are grown year after year. These practices dominate the landscape.

Organic corn was grown in about 160 farms with 11409 hectares (about 0.3% of total area in Minnesota), yielding average of 9.5 MT/ha. Organic corn production involves soil fertility management by using crop rotations over a four-year period with soybean, oats, vegetables and pastures. Synthetic pesticides are prohibited in organic systems and any pests are managed naturally by relying on natural biological control. Organic corn is primarily used as animal feed for livestock and poultry which are consumed by humans as meat products. Organic grains are also used for direct consumption and in various food products such as chips. Organic production depends on rotation to maintain soil health and livestock is increasingly becoming part of this rotation.

Organic farming system is a mixed production system where multiple organic crops are grown in rotation with livestock. The dominant corn production systems in Minnesota are summarised in Table S1 (see Supplementary Information).

### Framework for analysis of externalities in the corn systems

The TEEBAgriFood evaluation framework is founded on the concept of natural, social and human capital^37^. Globally, scientific literature has provided robust theoretical foundations to measure natural capital^5,38–40^, social capital^41^ and human capital^42^. These earlier works have been advanced during the development of TEEBAgriFood evaluation framework^3,5^. Its main goal is to evaluate all costs and benefits of agriculture by measuring natural, human and social capital in addition to produced capital in agriculture and food systems^37^. Measuring and accounting natural, human, and social capital stocks in agriculture and food systems contributes towards the wealth of nations^3^.

**Figure 3:**
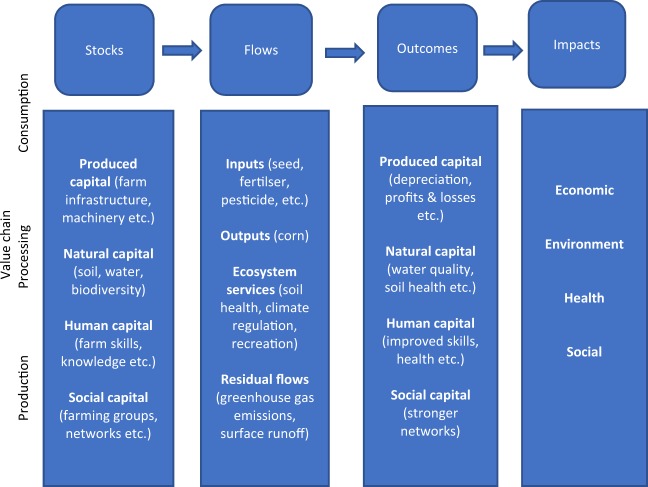
TEEBAgriFood evaluation framework as applied to the corn production systems (adapted from TEEB^5^).

Here, we focus on corn-based production systems in Minnesota, USA. Figure 3 shows four key elements of the TEEBAgriFood evaluation framework - stocks, flows, outcomes and impacts, which are being analysed in the corn systems^5^. Various elements of the framework are described below.

### Stocks

Stock is defined as a capital accumulated over time. To understand impacts and dependencies, it is important to understand capital base in agriculture. Therefore, four capitals are described below as they provide basis for the analysis in this study.

#### Produced capital

Produced capital used here is based on the concept measured in the Inclusive Wealth Report^43^ and defined by the TEEBAgriFood^5^. In corn production systems, produced capital includes farm produce, farm machinery and equipment, road networks, irrigation infrastructure, drying, storage facilities, etc. and financial capital such as farm loans, investment, insurance, etc^37^.

#### Social capital

Social capital includes all dimensions of social life, including interactions, networks, norms and trust, amongst members of society^44^. There are four key features of social capital; trust between members, opportunities for mutual benefits; set of common rules and interlinkages in groups and networks^45^. Social capital is essential to produce other forms of capital. It can be measured by assessing strengths in its structure, measuring relationships and shared values^37^. These can be measured by using various methods such as World Values Survey (WVS), Social capital index (SCI), and social survey^46^. In agriculture, farming group networks, partnerships with research and development, individual links, market linkages etc. form social capital.

#### Human capital

Human capital comprises of individual’s health, skills and knowledge that are essential for productive work^37^. Human capital considers that an investment in people is necessary to generate economic benefits^47^. It increases with improvements in the health, skills, experience and education of people. It is affected by the loss of skills and experience and by changes in human health. Main health risk pathways of corn production are through agri-environmental pollution of air, water and soil, mainly by synthetic fertilizers and herbicides.

#### Natural capital

Natural capital comprises of biodiversity and natural ecosystems that generates multiple benefits for the humanity in the form of ecosystem goods and services^39^.

### Flows

Flows are the benefits and impacts over a period of time during the use of various capitals. In agriculture and food systems, flows include farm produce, ecosystem services, and any residual flows such as pollution and greenhouse gas emissions. These are measured by using System of Environmental Economic Accounting Central Framework (SEEA^48^).

### Outcomes/change in wellbeing

Outcomes can be assessed by estimating the change in capital base, which can be either positive or negative. In this study, we report how the corn systems impacts on human wellbeing in Minnesota through change in four types of capital – produced, natural, social and human, in two diverse (GM and organic) corn production systems.

## Data Sources

### Produced, social and human capital (except health externalities)

We used statistical data available at the USDA National Agricultural Statistics Service Information to extract corn-related inputs and outputs data relevant to Minnesota. This was supplemented by relevant data from scientific literature and various health and environmental reports. We focused on Minnesota to examine two diverse corn production systems, therefore, we relied on data from Minnesota State. Corn data from the production year 2017 is presented for the Minnesota state to compare total acreage, production in metric tonnes (MT), market price (in 2017) per MT for both GM and organic corn. However, the comparison of farm expenditure is based on the USDA ERS data from 2010^49^, where average data from two corn regions – Heartland and Northern crescent is presented in absence of current estimates (adjusted to 2017 USD).

### Natural capital

We provide an estimate of the benefits and costs associated with corn production in terms of impacts of corn production on climate change, water quality, air quality, and soil quality, in addition to the benefit of crop production. For each indicator, we rely primarily on published studies that have assessed environmental and economic impacts of corn production in the Upper Midwestern U.S.

Economic value of provisioning ecosystem services (corn grains), was estimated from the annual U.S. Department of Agriculture market value of corn grain produced in Minnesota (2017 USD). We took the average of the last 20 years (1997–2017) and adjusted each year for inflation using the Bureau of Labor Statistics Consumer Price Index.

### Residual flows

Damages from climate change are globally distributed and emerge from emissions of greenhouse gases from different pathways associated with crop production. Here, we valued climate-related impacts of corn production by estimating CO_2_e emissions related to synthetic N fertilizer production and application and multiplying estimated emissions by the social cost of carbon. We use the 20-year average application rate to estimate the total amount of fertilizer applied to Minnesota corn systems^35^.

### Greenhouse gas emissions associated with synthetic N fertilizer production

We apply a production emissions factor of 0.004 MT CO_2_e per kg of N fertilizer^50^ to statewide application estimates for corn^35^. We used social cost of carbon at $42.55 per MT of CO_2_e emissions, assuming a 3% discount rate^51^.

### N_2_O emissions associated with synthetic N fertilizer use

We multiplied the social cost of N_2_O emissions from fertilizer application^52^ by the average annual N fertilizer application in Minnesota^35^. We used social cost of N at $0.235 per kg N emissions, assuming a 3% discount rate.

### Water quality

Agriculture is the dominant driver of water pollution in Minnesota, with the majority of nutrient export coming from agricultural production^53^. Agricultural pollutants include nutrients such as nitrogen and phosphorus, as well as sediment from runoff.

### Nitrogen

Nitrates pose a threat to drinking water quality and are the major driver of eutrophication in the Gulf of Mexico. Because of spatial heterogeneity in the risk of nitrates reaching drinking water sources and heterogeneous exposure of streams and rivers to nitrates, the impacts and associated costs of these externalities vary spatially. The impacts of nitrate contamination of drinking water can be estimated by the costs of various treatment options and applying a weighted cost function based on the observed adoption of those technologies as a proportion of total treatment^25^. Some studies include the cost of health impacts from increased nitrate consumption in the cost calculations, weighted by the no-treatment fraction^25^. We applied the median values of the social cost functions to the average annual state wide N application to corn in Minnesota^35^. We used $0.075 median cost of exposure of NO_3_- per kg N.

### Phosphorus

The externalities of phosphorus pollution are primarily from negative impacts to lake and river water quality. In large enough quantities, phosphates cause lakes and other bodies of water become eutrophic, a state dominated by excessive plant growth and algal blooms. Corn production contributes to phosphorus pollution in Minnesota, thus changes in agricultural policies or associated land uses that affect phosphorus export will increase or decrease value attributed to clean water accordingly. Previous studies examined the social cost of phosphorus pollution using hedonic^54^ and recreation travel cost^55^ approaches. However, because travel cost and hedonic methods rely on understanding the biophysical responses of individual lakes and local market conditions that cannot be extrapolated statewide, we did not apply them to the water quality impacts of corn production.

We use estimates of P export from cropland modelled by the Minnesota Pollution Control Agency^56^ and weighted those by the proportion of cropland that is used for corn production. We multiply this by a shadow cost of P ($55.43 per kg P) export estimated by the Wisconsin Department of Natural Resources^57^.

### Air quality

Increases in particulate matter and associated health impacts are a global consequence of fertilizer application. We used an atmospheric transport model^58^ to estimate atmospheric concentrations of PM2.5 emissions from corn production and resulting health impacts. These impacts vary spatially, depending on pollutant concentrations and number of people affected. We used the median value of $0.55 per kg N and multiplied it by the average amount of N application in Minnesota^35^.

### Soil loss

Erosion driven by wind or water reduces the quality and productive potential of soil, and eroded sediment in waterways can damage infrastructure and fisheries. We used long- term measurements of water and wind erosion on cultivated land in Minnesota^35^ and multiplied soil quantity lost by costs ($5.93 per MT of corn, estimates for corn belt region)^59^.

### Non-financial health costs

Non-financial costs supplement classical financial costs. Financial costs are easy to account for because their value depend on the market. In contrast, non-financial costs have their value determined by a physical net worth that has no active market of buyers or sellers. For example, business organisations in European Union are required to publish reports on their social and environmental impacts including treatment of employees, human rights conditions, anti-corruption, diversity information etc. according to the EU rules (Directive 2014/95/EU). We use three-stage well-being valuation method (Methodology for Valuing the Agriculture and the wider food-system Related Cost of Health, MARCH)^4^ to estimate the non-financial health costs of corn production in Minnesota. The impact of corn production on health is monetised by equating the change in well-being from a marginal increase in corn production to the change in income required to yield an equivalent change in well-being. This method is used to measure the association between corn production near individuals’ residences and their general health in Minnesota. We have linked land use data to measures of general health from the Gallup Daily tracking survey. Well-being valuation method is then used to monetize the general health impact of corn production. This method works by calculating the amount of money that would induce an impact on life satisfaction equivalent to the impact of 1% increase in corn production intensity close to individuals’ residence. Thus, this model finds the implied non-financial health costs of corn production (see Supplementary Information for details).

## Supplementary information

Supplementary Information.

## Data Availability

The data is included in the manuscript and the supplementary information.
