# Supplementary material for: Evaluating the holistic costs and benefits of corn production systems in Minnesota, US
Source: Sci Rep. 2020 Mar 3;10:3922. doi: 10.1038/s41598-020-60826-5 (PMC7054304; doi:10.1038/s41598-020-60826-5)
Supplement: Supplementary file 1 — Supplementary Information. [file 41598_2020_60826_MOESM1_ESM.pdf]

Supplementary Information

Evaluating the holistic costs and benefits of corn production systems in Minnesota, US

Harpinder Sandhu[1,2*], Nadia El-Hage Scialabba[3], Chris Warner[4], Fatemeh Behzadnejad[4], Kieran Keohane[4], Richard Houston[4], Daniel Fujiwara[4],

[1] School of Natural and Built Environments, University of South Australia
[2] College of Science and Engineering, Flinders University, Australia.
[3] Food and Agriculture Organization of the United Nations, Rome, Italy.
[4] Simetrica, London, UK

* Correspondence to Harpinder.Sandhu@unisa.edu.au

1. Farming systems

The dominant corn production systems in Minnesota are summarised in Table S1.

Table S1 Farming systems in Minnesota. Source (USDA ERS 2018).

	**Corn system**	**Rotation**	**Practices**	**Tillage**
Very large farms	Genetically Modified corn	Soybean	Conventional	Minimum tillage
Very large farms	Hybrid corn	Soybean	Conventional	Conventional
Large farms	GM/Hybrid corn	Soybean	Sustainable practices	Strip-tillage
Large farms	GM/Hybrid corn	Soybean/ Alfalfa	Sustainable practices	Cover cropping
Farming occupation farms/low sales	Mixed cropping organic corn	Mixed crops – soybean, oats, barley, pastures, vegetables, fruits	Organic management	Minimum tillage/conventional tillage/rotational grazing

Where,
Very large farms: Farms with gross cash farm income (GCFI) of $5,000,000 or more
Large farms: Farms with GCFI between $1,000,000 and $4,999,999
Farming-occupation farms. Small farms whose principal operators report farming as their primary occupation.
Low-sales farms: GCFI less than $150,000.
Moderate-sales farms: GCFI between $150,000 and $349,999
Midsize family farms: GCFI between $350,000 and $999,999

2. Valuation of non-financial health costs associated with corn production

Data sources

The three main sources of data for valuation of non-financial health costs are:

1. Gallup daily tracking survey: Gallup conducts a daily survey administered to 1,000 U.S. adults on topics pertaining to various demographic, political, economic, and well-being themes. For this study, we pool 10 years of cross-sectional microdata from the Gallup Daily Survey for the State of Minnesota from 2008 to 2017. The Gallup Daily Survey is designed to be representative at the state-level. It also includes a ZIP-code identifier for each individual which we used as a proxy for where they live.
2. Corn production intensity: We used the Cropland Data Layer (CDL; National download for 2008-2017 is available at https://www.nass.usda.gov/Research_and_Science/Cropland/Release/index.php . The remaining data for 2006 and 2007 was downloaded from CropScape - https://nassgeodata.gmu.edu/CropScape/) published by the USDA National Agricultural Statistics Service (NASS) to calculate land use intensity for corn and non-corn production, which is calculated as the proportion of a survey respondents' surroundings used in corn and non-corn production. The CDL is satellite data which measures land use at a 30m by 30m scale for the years 2006-2017 for the entire state of Minnesota. We take ZIP code centroids (a geographic centroid is the mean position of all the points within the area of the ZIP code), constructed areas of varying radius around them (circular 'buffers') and then calculated the proportion of the buffer used for corn and non-corn production as a measure for the intensity of land use in corn and non-corn production in an individual's surroundings. For example, a circular buffer of 5km radius (with an area of approximately 78km^2) around a ZIP code would be considered 10% corn if 7.8 kilometres of the buffer was used for corn production. This approach allows us to calculate land use intensity for corn and non-corn production (henceforth referred to as 'corn' and non-corn intensity) for all 863 ZIP codes in Minnesota. Merging the ZIP code measures of corn and non-corn intensity with the Gallup respondents' ZIP code and year of survey obtains the area used for corn, and the area used for all other crops in each buffer.
3. Organic corn production data: The CDL data set described is not able to distinguish between organic and non-organic corn.

Methodology

To measure the impact of corn production on health, we followed the well-being valuation method explained in MARCH (FAO, 2017), which offers an alternative to the Quality-Adjusted Life Years (QALYs) approach of valuing the non-financial costs of health. To use this method, we first estimated the impact of a non-market good (in this case corn intensity), income, and other determinants of wellbeing on measures of subjective well-being (SWB), such as life satisfaction. Thus, as displayed in Figure S1, the wellbeing valuation method measures two effects: the impact of the non-market good on SWB (β_C) and the impact of income on SWB (β_M).

Consequently, using the estimated impacts of income and the non-market good, we assessed the monetary value of the non-market good. This monetary value shows how

much an individual would have to be compensated to return their wellbeing to its original level (the status quo without the non-market good).

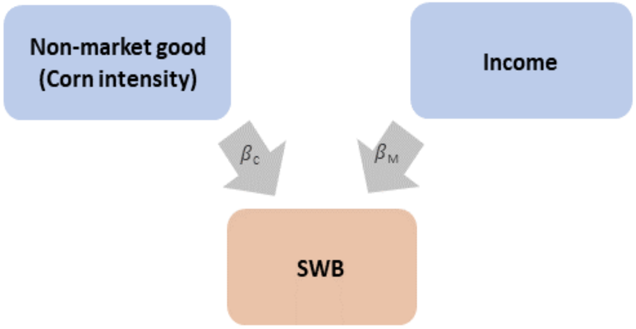

Figure S1 Graphical representation of the WV method for valuing corn intensity as a non-market good.

In this study, the non-market good being valued is corn intensity in the proximity of where individuals live and the measure of SWB is their life satisfaction (In the Gallup data, life satisfaction is measured by the Cantril ladder scale which poses the following question: "Please imagine a ladder with steps numbered from zero at the bottom to ten at the top. Suppose we say that the top of the ladder represents the best possible life for you and the bottom of the ladder represents the worst possible life for you. If the top step is 10 and the bottom step is 0, on which step of the ladder do you feel you personally stand at the present time?). The monetary value obtained through the wellbeing valuation method is the well-being effect of corn intensity through its impacts on health. This monetary value can also be interpreted as the (non-financial) costs of health associated with corn intensity. Note that the WV method does not account for any health impact caused by the consumption of products containing corn (as corn intensity can be assumed to be independent of the amount of corn consumed, not including corn consumption in the model does not bias our estimates).

Variables

We used following variables suggested in Fujiwara and Campbell (2011):

- Age
- Gender
- Marital status
- Educational status
- Employment status and income
- Religious affiliation
- Number of children
- Geographic region
- Urbanization
- Local environment conditions
- Year

Monetary value

The valuation of non-financial health costs of corn production is based on the well-being valuation method. First, we estimate the impact of corn intensity (δ) on general health.

From Table S2, estimating the model using 5 km and 10 km buffers results in a value for δ of -0.0021 and -0.0025, respectively (statistically significant at the 5% level). The sign of δ indicates a negative association between experienced corn intensity and an individual's health. Based on these estimations, the negative impact is statistically significantly higher in the 10 km buffer compared with 5 km buffer. As the geographical area of a ZIP code is large (The average, minimum, and maximum values for the ZIP code geographic areas in Minnesota are 244 km^2, 0.15 km^2, and 289 km^2), particularly in rural areas, the corn intensity measured in a 10 km buffer more precisely represents the intensity experienced by individuals throughout the ZIP code area.

The value of δ implies that an increase in corn intensity in a 10 km buffer by 1% will decrease general health by 0.0025 points (where general health is measured by a 1-5 scale). Increasing the corn intensity by 9.74%, which is the average land used for corn production in 10 km buffer in our sample, implies a decrease of 0.024 points in general health. In other words, going from no corn production to the average level of corn production (holding other factors in the model constant) implies a 0.67% decrease in general health (relative to average levels of general health).

Consequently, we apply the wellbeing valuation method to estimate the non-financial health costs of corn intensity in the respondents' surrounding area. The non-financial health costs associated with a 1% increase in corn intensity in the vicinity of an individual's residence is $20.7 per year in the 5km buffer and $24.7 per year in the 10 km buffer. These results are based on Minnesota average household income 2016 which, according to US Census Bureau, was $83,100.

Table S2Estimated per-person association of corn intensity and general health and valuation of non-financial health costs.

	**With a 5 km buffer around ZIP-code centroid**	**With a 10 km buffer around ZIP-code centroid**
Association of corn intensity with general health	-0.0021**	-0.0025***
Annual value of health costs associated with an additional 1% corn intensity per person	$20.70	$24.70
Annual value of health costs per person associated with corn intensity=9.74% (the average intensity for Minnesota in the sample)	$180.10	$240.30
** Statistically significant at 5% level		
*** Statistically significant at 1% level		

Our analysis in this part reveals that although 26% of the sample have at least one organic farm with some corn production in their ZIP code, organic corn farms are particularly small on average comprising 0.3% of total land used for corn production. This means that the proportion of sample likely to be impacted on by an organic corn farm is much lower than that of a non-organic farm. The relatively lower size of organic corn farms might be an

important issue in identifying the health impacts of organic versus non-organic corn production in case that we have access to a more complete and detailed data set.

Aggregating Health Costs: To calculate annual non-financial health costs associated with corn production in Minnesota, we follow the following steps:

1. Using the wellbeing valuation method, we obtain the monetary value of the average health costs on individuals of a 1% increase in the intensity of corn production in their respective ZIP code.
2. Using data from the United States Census Bureau, we find the population for each county in Minnesota. The population of each county is then multiplied by the health costs obtained in step 1. This will give us the health costs per 1% corn intensity in each county. Note that our estimates are based on a sample of respondents aged over 18. If we assume that individuals aged under 18 are not affected differently by corn intensity, we multiply the whole population of each county to the health costs per individual. For example, for Dakota county, with a total population of 421,751, the annual health costs associated with a 1% increase in land used for corn in a 10 km buffer will be: 421,751 × $24.7 = $ 10.42 million.
3. To calculate the health costs of corn production per county, we multiply the number obtained in step 2 by average corn intensity for the county. For example, In Dakota county, the average corn intensity in a 10 km buffer is 8.63%, so the annual health costs of corn production are: 10.42 × 8.63 = $ 89.90 million
    a. Table S3 shows 40 counties with the highest health costs of corn production based on relative intensity in a 10 km buffer.
4. Finally, we find the total health costs in Minnesota by aggregating all counties health costs. Based on our model, the annual non-financial health costs of corn production in Minnesota are about $ 1.3 billion (approximately $233 per capita). This is broadly aligned with the costs obtained in MARCH (FAO, 2017). In this study, the annual non-financial health costs of the UK food system for different health problems were between $107 per capita and $1372 per capita.

Table S3 Non-financial health costs associated with corn production for each county in Minnesota.

**Rank**	**County**	**County Population**	**Average corn intensity in 10 km buffer (%)**	**Annual health Costs based on 10 km Buffer (Million $)**
1	Dakota	421,751	8.63	89.9
2	Olmsted	154,930	17.57	67.2
3	Stearns	157,822	17.17	66.9
4	Wright	134,286	15.37	51
5	Blue Earth	66,973	27.08	44.8
6	Washington	256,348	7.01	44.4
7	Rice	65,968	27.03	44
8	Scott	145,827	11.52	41.5
9	Carver	102,119	14.53	36.7
10	Mower	39,566	33.87	33.1
11	Steele	36,887	35.56	32.4

12	Hennepin	1,252,024	1.04	32.1
13	McLeod	35,884	34.85	30.9
14	Kandiyohi	42,743	29.05	30.7
15	Nicollet	33,966	35.1	29.4
16	Goodhue	46,304	23.55	26.9
17	Lyon	25,831	41.78	26.7
18	Freeborn	30,535	33.91	25.6
19	Nobles	21,944	41.97	22.8
20	Brown	25,194	36.17	22.5
21	Benton	39,937	22.66	22.4
22	Martin	19,850	44.53	21.8
23	Sherburne	94,570	8.95	20.9
24	Clay	63,569	13.12	20.6
25	Le Sueur	28,111	27.64	19.2
26	Dodge	20,762	37.18	19.1
27	Waseca	18,787	40.54	18.8
28	Otter Tail	58,345	11.91	17.2
29	Anoka	351,373	1.95	16.9
30	Meeker	23,131	29.04	16.6
31	Sibley	14,869	41.79	15.3
32	Renville	14,645	41.74	15.1
33	Redwood	15,272	39.68	15
34	Faribault	13,784	43.76	14.9
35	Fillmore	20,980	23.58	12.2
36	Watonwan	10,840	45.48	12.2
37	Chisago	55,308	8.74	11.9
38	Winona	50,873	9.14	11.5
39	Isanti	39,582	11.68	11.4
40	Wabasha	21,608	21.14	11.3
	First 40 counties	4,073,098	11.17	1123.8
	All other counties	1,503,508	4.75	176.53
	Minnesota	5,576,606	9.74	1300.33

References cited

- FAO, 2017. Methodology for valuing the Agriculture and the wider food system Related Costs of Health (MARCH). Food and Agricultura Organization of the United Nations, Rome.

- Fujiwara D and Campbell R, 2011. Valuation Techniques for Social Cost-Benefit Analysis: Stated Preference, Revealed Preference and Subjective Well-Being Approaches. A discussion of the Current issues. HM Treasury. Department of Work

and Pensions. July 2011. Fukuyama F, 1996. Trust: The Social Virtues and the Creation of Prosperity. Harmondsworth: Penguin Books.

- USDA ERS, 2018. United States Department of Agriculture Economic Research Service. Agricultural Productivity in the U.S. https://www.ers.usda.gov/data-products/agricultural-productivity-in-the-us/

3. Social networks in Minnesota

Table S4 Social networks available to growers in Minnesota. X means available.

	Network	Dimension	In GM corn	In Organic	Informal/Formal/Transactional
Government	US Department of Agriculture	Relational	X	X	Informal
	Minnesota Department of Agriculture	Relational	X	X	Informal
	US Department of Agriculture-Rural Development	Relational	X	X	Informal
	Farm service in counties	Relational	X	X	Informal
	American Farm Bureau	Relational	X	X	Informal
	Minnesota Farm Bureau	Relational	X	X	Informal
	National Farmers' Union	Relational	X	X	Informal
	Minnesota Extension Service	Relational	X	X	Informal
	Agricultural Utilization Research Institute	Relational	X	X	Informal
	Center for Farm Financial Management	Relational	X	X	Informal
	Minnesota Agriculture Education Leadership Council	Relational	X	X	Informal
	USDA Farm Service Agency (FSA)	Relational	X	X	Formal
	USDA Natural Resources Conservation Service	Relational	X	X	Formal
	Minnesota	Relational	X	X	Formal
	USDA Animal and Plant Health Inspection Service	Relational	X	X	Formal

	USDA Risk Management Agency	Relational	X	X	Formal
	Farm Service Agency/Board of Water and Soil Resources	Relational	X	X	Formal
	Minnesota Conservation Reserve Enhancement Program (MN CREP)	Relational	X	X	Formal
	Reinvest In Minnesota Reserve Program (RIM)	Relational	X	X	Formal
	Conservation Cost-Share Program	Relational	X	X	Formal
	Agriculture BMP Loan Program (AgBMP)	Relational	X	X	Formal
	Minnesota Agricultural Water Quality Certification Program (MAWQCP)	Relational	X	X	Formal
	Environmental Quality Incentives Program (EQIP)	Relational	X	X	Formal
	Conservation Stewardship Program (CSP)	Relational	X	X	Formal
	Agricultural Conservation Easement Program (ACEP)	Relational	X	X	Formal
	Faribault County Soil/Water Clean Water Partners Cover Crop Assistance Program	Relational	X	X	Formal
Research	The Minnesota Institute for	Relational	X	X	Informal

	Sustainable Agriculture				
	University of Minnesota Extension	Relational	X	X	Informal
	University of Minnesota: Department of Applied Economics	Relational	X	X	Informal
	University of Minnesota: Department of Family Social Science: Rural MN Life	Relational	X	X	Informal
	University of Minnesota: College of Food, Agriculture and Natural Resource Sciences	Relational	X	X	Informal
	Economic Research Service, USDA	Relational	X	X	Informal
	Center for Transportation Studies	Relational	X	X	Informal
	Kellogg Collection of Rural Community Development Resources	Relational	X	X	Informal
	National Sustainable Agriculture Information Service	Relational		X	Informal
	Sustainable Agriculture Research and Education	Relational		X	Informal
	Rural Policy Research Institute			X	Informal
Farming/environment groups	Minnesota Farmers Union	Structural/cognitive/relational	X		Formal
	Cover crop group	Structural/cognitive/relational	X		Informal
	Strip tillage group	Structural/cognitive/relational	X		Informal

	Soil health partnership	Structural/cognitive/relational	X	X	Informal
	The National Corn Growers Association	Structural/cognitive/relational	X		Informal
	Minnesota Corn Growers Association	Structural/cognitive/relational	X		Informal
	The Land Stewardship Project	Relational	X	X	Informal
	The Sustainable Farming Association of Minnesota	Structural/cognitive/relational		X	Informal
	MOSES-Midwest Organic and Sustainable Education Services	Structural/cognitive/relational		X	Informal
	ALBA-Agricultural and Land Based Association	Structural/cognitive/relational		X	Informal
	Attra- National Sustainable Agricultural Information Services	Structural/cognitive/relational		X	Informal
	Farmers' Legal Action Group	Structural/cognitive/relational	X	X	Informal
	Local Dirt	Structural/cognitive/relational	X	X	Informal
	Renewing the Countryside	Structural/cognitive/relational	X	X	Informal
	American Farmland Trust	Structural/cognitive/relational	X	X	Informal
	Smart Communities Network	Structural/cognitive/relational	X	X	Informal
	Minnesota Environmental Initiative	Structural/cognitive/relational	X	X	Informal
	Minnesota Land Trust	Structural/cognitive/relational	X	X	Informal
	Mississippi Headwaters Board	Structural/cognitive/relational	X	X	Informal
	1000 Friends of Minnesota	Structural/cognitive/relational	X	X	Informal
	Northern Prairie Wildlife	Structural/cognitive/relational	X	X	Informal

	Research Center				
	SmartGrowth	Structural/cognitive/relational	X	X	Informal
	Sprawl Watch Clearinghouse	Structural/cognitive/relational			Informal
Businesses	Agri-chemical dealers	Structural/relational	X		Transactional
	Seed dealers	Structural/relational	X	X	Transactional
	Ethanol plant cooperatives	Structural/relational	X		Transactional
	Corn buyers	Structural/relational	X	X	Transactional
	Insurance companies/agents	Structural/relational	X	X	Transactional
	Banks	Structural/relational	X	X	Transactional
	Cooepratives	Structural/relational	X		Transactional
	Farm machinery companies	Structural/relational	X	X	Transactional
	Organic certification	Structural/relational		X	
Individuals	Neighbours/friends	Cognitive/relational	X	X	Personal
	Rural town/community	Cognitive/relational	X	X	Personal
Foundations and Non-profits	Blandin Foundation	Cognitive/relational	X	X	Informal
	McKnight Foundation	Cognitive/relational	X	X	Informal
	Minnesota Council on Foundations	Cognitive/relational	X	X	Informal
	Minnesota Council of Non-profits	Cognitive/relational	X	X	Informal
	Bush Foundation	Cognitive/relational	X	X	Informal
	Center for Rural Strategies	Cognitive/relational	X	X	Informal
	Farm Foundation	Cognitive/relational	X	X	Informal
